# Modeling coding-sequence evolution within the context of residue solvent accessibility

**DOI:** 10.1186/1471-2148-12-179

**Published:** 2012-09-12

**Authors:** Michael P Scherrer, Austin G Meyer, Claus O Wilke

**Affiliations:** 1 Center for Computational Biology and Bioinformatics, Institute for Cellular and Molecular Biology, and Section of Integrative Biology, The University of Texas at Austin, Austin, TX 78712, USA

## Abstract

**Background:**

Protein structure mediates site-specific patterns of sequence divergence. In particular, residues in the core of a protein (solvent-inaccessible residues) tend to be more evolutionarily conserved than residues on the surface (solvent-accessible residues).

**Results:**

Here, we present a model of sequence evolution that explicitly accounts for the relative solvent accessibility of each residue in a protein. Our model is a variant of the Goldman-Yang 1994 (GY94) model in which all model parameters can be functions of the relative solvent accessibility (RSA) of a residue. We apply this model to a data set comprised of nearly 600 yeast genes, and find that an evolutionary-rate ratio *ω *that varies linearly with RSA provides a better model fit than an RSA-independent *ω *or an *ω *that is estimated separately in individual RSA bins. We further show that the branch length *t* and the transition-transverion ratio *κ *also vary with RSA. The RSA-dependent GY94 model performs better than an RSA-dependent Muse-Gaut 1994 (MG94) model in which the synonymous and non-synonymous rates individually are linear functions of RSA. Finally, protein core size affects the slope of the linear relationship between *ω *and RSA, and gene expression level affects both the intercept and the slope.

**Conclusions:**

Structure-aware models of sequence evolution provide a significantly better fit than traditional models that neglect structure. The linear relationship between *ω *and RSA implies that genes are better characterized by their *ω *slope and intercept than by just their mean *ω*.

## Background

Substitution patterns in protein-coding genes are shaped by the 3-dimensional structure of the expressed proteins. To account for this influence of structure on sequence evolution, evolutionary biologists increasingly aim to combine sequence analysis with structural information or to develop models of sequence evolution that incorporate structural features of the expressed protein. Some authors calculate amino-acid substitution matrices as a function of protein structure [1,2] or correlate sequence variability in alignments with structural features [3,4]. Others subdivide proteins into broad categories by solvent exposure (buried/exposed) or secondary structure (*α*-helix, *β*-sheet, etc.) and then use standard maximum-likelihood models of sequence evolution to infer evolutionary rates as a function of structural features [5-9]. Some authors employ more complex methods that allow for non-independence among sites, and use energy functions to model how substitutions at one site influence substitutions at others [10-13]. Finally, a few groups have attempted a variety of other approaches to link sequence variability with protein structure [14-17].

These various analyses differ in their specific results as well as in the approaches taken. However, one pattern consistently emerges: Residues in the core of proteins are more conserved than residues on the surface. This finding agrees with our understanding of protein biochemistry. Substitutions in the core of a protein are more likely to disrupt fold stability than substitutions on the surface, and the loss of the structural integrity of a protein is frequently the underlying cause of loss of function [18,19]. Further, the observed relationship between residue buriedness and evolutionary conservation seems surprisingly simple. When evolutionary rate is plotted as a function of relative solvent accessibility (RSA, a number between 0 and 1 measuring how exposed a residue is to the solvent surrounding the protein), one finds a near-perfect linear relationship [9,20].

Inspired by the observed linear relationship between evolutionary conservation and RSA, we here take the standard Goldman-Yang model of coding-sequence evolution (GY94, [21]) and introduce to it a dependency of the model parameters on RSA. We find that the RSA-dependent GY94 model provides a substantially better fit to yeast sequence data than the standard, RSA-independent model. We further find that for several model parameters, a simple, linear dependency on RSA provides the best fit. In particular, the ratio of non-synonymous to synonymous evolutionary rates *ω* is a linear, increasing function of RSA. Thus, we can characterize protein evolutionary rates by the slope and intercept of the *ω*–RSA relationship rather than by just a single *ω* value. We show that slope and intercept of the *ω*–RSA relationship vary among proteins with different structures or different expression levels.

## Results

### An RSA-dependent Markov model of coding-sequence evolution

Previous works assessing the relationship between evolutionary rate and RSA subdivided sites into groups with comparable RSA and then calculated evolutionary rates separately for each group [9,20]. This approach yields a set of independent evolutionary-rate estimates that can be plotted against representative RSA values for each group. While this approach has provided valuable new insight, it is not satisfactory from a methodological perspective. First, some model parameters (such as parameters describing the nucleotide-level mutation process, e.g. the transition–transversion bias) could be conserved among groups. Yet they are estimated individually for each group. Second, a consistent framework for hypothesis testing is lacking. For example, in order to test whether evolutionary rates vary linearly with RSA, one would have to do a regression analysis on the previously estimated rates. In this regression analysis, sample size corresponds to the number of RSA groups rather than to the number of sites in the original data set. Consequently, the *P* value resulting from the regression would likely be incorrect.

To resolve these shortcomings, we developed a variant of the GY94 model [21] in which model parameters are functions of RSA. We write the infinitesimal generator *Q *= (*Q*_*ij*_) of the Markov process describing the substitution process as (for *i *≠* j*)

(1) $$Q_{\text{ij}}=\left\{\begin{array}{rl} 0, & \text{if more than one nucleotide change}\\ \Pi_{j}, & \text{if synonymous transversion}\\ \kappa (r)\Pi_{j}, & \text{if synonymous transition}\\ \omega (r)\Pi_{j}, & \text{if nonsynonymous transversion}\\ \kappa (r)\omega (r)\Pi_{j}, & \text{if nonsynonymous transition} \end{array}\right),$$

where *κ* is the ratio of transitions to transversions, *ω *is the ratio of the nonsynonymous to synonymous substitution rates, and *r* stands for the RSA of a site. The indices *i* and *j* run over all 61 sense codons, and *Π*_*j *_is the frequency of codon *j*. (We do not estimate site-specific codon frequencies). The finite-time transition matrix is given by

(2) P=exp[t(r)Q],

where *t* corresponds to evolutionary time, in arbitrary units. The parameter *t* measures the branch length in the phylogenetic tree; it is broadly related to the rate of synonymous substitutions. On first glance, it might be surprising that we allow *t* to vary with RSA. However, as we will see below, models with site-dependent *t* fit the data better than models with a single *t* across all sites. The reason for the improved fit is that RSA influences both amino-acid level processes and nucleotide-level processes.

We implemented this model in the phylogenetic modeling language HyPhy [22]. One problem we faced was that HyPhy does not allow a continuous co-variable (such as *r*) in the model matrix. To overcome this technical problem, we binned RSA values into *n* bins and represented all RSA values within bin *k* by the bin mid-point, which we denote by *r*_*k*_. In this way, we approximate a single matrix *Q*(*r*) that changes continuously with *r* by a set of *n* discrete matrices *Q*_*k *_=* Q*(*r*_*k*_), with *k* = 1,…,*n*. HyPhy allows us to simultaneously fit multiple discrete matrices, and it also allows us to share parameters among these matrices. In the limit of large *n*, our discretized model converges to the model that is continuous in *r*.

**Figure 1:**
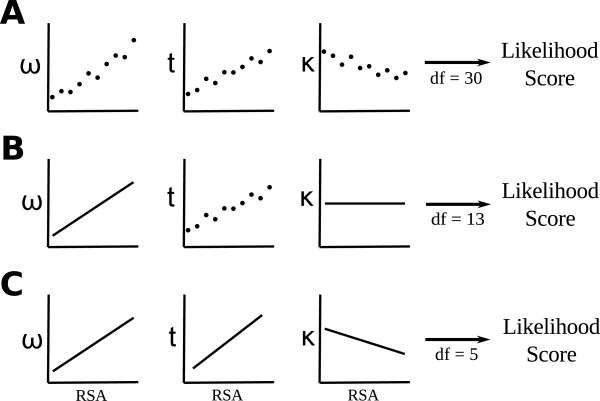
**Examples of RSA-dependent sequence-evolution models considered.** All models have three parameters, evolutionary-rate ratio *ω*, branch length *t*, and transition–transversion ratio *κ*. All three parameters can be estimated as an individual value within each RSA bin (per-bin), as a linear function of RSA (linear), or as a constant across all RSA values (constant). The examples here are illustrated for *n *= 10 RSA bins. (**A**) All parameters are estimated per-bin. (**B**) *ω*is estimated as linear function, *t* is estimated per-bin, and *κ *is estimated as a constant. (**C**) All paramters are estimated as linear functions.

Our model contains three fitted parameters: *ω*(*r*), *κ*(*r*), and *t*(*r*). For each parameter, we considered three types of RSA dependency. First, a parameter can be constant, i.e., not actually depend on RSA. In this case, we have *ω*(*r*) =* ω*_0_, *κ*(*r*) =* κ*_0_, or *t*(*r*) =* t*_0_. Second, a parameter can be a linear function of RSA. In this case, we have *ω*(*r*) =* ω*_0_ + *ω*_1_*r*, *κ*(*r*) =* κ*_0_ + *κ*_1_*r*, or *t*(*r*) =* t*_0_ + *t*_1_*r*. (But note that we actually use only *n* discrete RSA values *r*_*k*_, because of the binning procedure). Finally, we can allow for separate *ω*, *κ*, and *t* values in each bin. (We refer to this case as *per-bin* parameter estimation). In this case, we fit *n* distinct *ω* values, one for each bin (which we refer to as $\omega_{r_{k}}$), and likewise for *κ* and *t*. Figure 1 illustrates the various modeling choices for *ω*, *κ*, and *t*, in various combinations.

### A linear RSA ependency for all estimated parameters provides the best model fit

We fitted our model to a data set of yeast sequences with available structural information. We identified 587 *Saccharomyces cerevisiae* genes with known ortholog in *Saccharomyces paradoxus* and with a representative structure in the Protein Data Bank (PDB). We calculated RSA for each site as described [7]. Unless noted otherwise, we used *n *= 20 evenly-spaced RSA bins.

Since we considered three different functional forms of RSA dependence (constant, linear, and per-bin) for each of the three parameters *ω*, *κ*, and *t*, we had 27 possible models. We fit all these models to our data set and ranked them by their Akaike Information Criterion (AIC [23,24]). Results for all models are shown in Table 1. The top-scoring model was one in which *ω* and *t* depended linearly on RSA while *κ*was estimated per bin. The differences in AIC were quite substantial among models, and the top-scoring model was clearly better than the next-best model (in which all parameters were estimated as linear functions).

**Table 1:** Fitted models, in order of ascending AIC

***ω***	***t***	***κ***	**ln*L***	***df ***	**AIC**	***t *slope**	***κ *slope**
linear	linear	per-bin	−839713.86	24	1679476	+	−
linear	linear	linear	−839736.74	6	1679485	+	−
per-bin	linear	per-bin	−839701.37	42	1679487	+	−
per-bin	linear	linear	−839722.37	24	1679493	+	−
linear	per-bin	linear	−839723.27	24	1679495	+	−
linear	per-bin	per-bin	−839707.75	42	1679499	+	−
per-bin	per-bin	linear	−839710.08	42	1679504	+	−
per-bin	per-bin	per-bin	−839694.42	60	1679509	+	−
linear	constant	linear	−839757.23	5	1679524	0	−
per-bin	constant	linear	−839740.64	23	1679527	+	−
linear	constant	per-bin	−839742.62	23	1679531	0	−
per-bin	constant	per-bin	−839727.25	41	1679537	0	−
linear	linear	constant	−839825.99	5	1679662	−	0
per-bin	linear	constant	−839809.70	23	1679665	−	0
linear	per-bin	constant	−839817.06	23	1679680	−	0
per-bin	per-bin	constant	−839800.41	41	1679683	−	0
linear	constant	constant	−839867.98	4	1679744	0	0
per-bin	constant	constant	−839856.43	22	1679757	0	0
constant	linear	per-bin	−840468.84	23	1680984	+	−
constant	per-bin	per-bin	−840459.99	41	1681002	+	−
constant	per-bin	linear	−840479.14	23	1681004	+	−
constant	linear	linear	−840524.57	5	1681059	+	−
constant	linear	constant	−840697.41	4	1681403	+	0
constant	per-bin	constant	−840688.35	22	1681421	+	0
constant	constant	linear	−840738.77	4	1681486	0	−
constant	constant	constant	−840740.37	3	1681487	0	0
constant	constant	per-bin	−840726.86	22	1681498	0	0

In general, we found that all parameters varied significantly with RSA. The top eight models did not contain a single model in which even one parameter was constant over RSA. This result shows that it is not sufficient to just make *ω* a function of RSA, the transition–transversion bias *κ *and the branch-length *t* also depend on RSA. Among the models with constant parameters, models with constant *t* ranked the highest. Models with constant *ω *ranked consistently the lowest. This result highlights the strong dependency of amino-acid substitution patterns on RSA.

Whenever the transition–transversion bias *κ *was allowed to vary with RSA, either linearly or per-bin, we found that it generally had a negative slope (decreased with increasing RSA). The branch length *t* tended to have a positive slope (increased with increasing RSA), unless *κ* was made constant, in which case *t* assumed a negative slope (Table 1).

**Figure 2:**
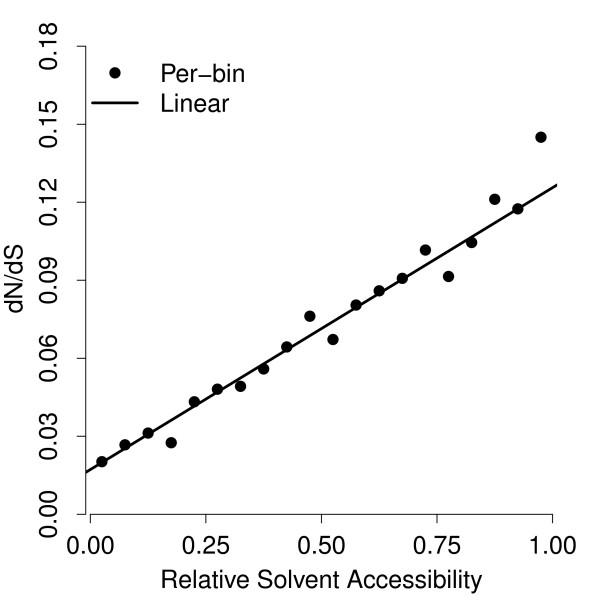
**Evolutionary-rate ratio increases linearly with RSA.** The solid line shows *ω *=* dN*/*dS *versus RSA as estimated by the best model (linear *ω*, linear *t*, per-bin *κ*). The dots show the same for the best model with per-bin *ω *(which has linear *t* and per-bin *κ*). Both models are consistent with each other and strongly support a linear relationship between *ω *and RSA.

Figure 2 shows *ω *as a function of RSA as estimated for the overall best model (with linear *ω* and *t* and per-bin *κ*) and, for comparison, for the overall best model with per-bin *ω *(with linear *t* and per-bin *κ*). We see that the estimates from both models are highly consistent with each other, and that the per-bin estimates strongly support a linear relationship between *ω *and RSA.

To assess the effect of the binning procedure on model estimation, we re-fitted the fully linear model (with linear *ω*, *κ*, and *t*) using different numbers of bins, from *n *= 4 to *n *= 20. Parameter estimates were nearly independent of *n* and varied smoothly in *n* (Table 2). We obtained similar results when we used a model with linear *ω* and *t* and per-bin *κ *(data not shown).

**Table 2:** Effect of the number of bins on parameter estimates

***n***	***ω***_**0**_	***ω***_**1**_	***t***_**0**_	***t***_**1**_	***κ***_**0**_	***κ***_**1**_	**ln*L***		
4	0.1205	0.0106	0.7110	2.4706	-2.5487	5.3465	-839824.56		
5	0.1208	0.0116	0.6967	2.4734	-2.5948	5.3547	-817178.82		
6	0.1162	0.0135	0.7012	2.4828	-2.5361	5.3136	-839781.41		
7	0.1149	0.0143	0.7034	2.4849	-2.5102	5.2976	-839764.54		
8	0.1138	0.0148	0.7269	2.4805	-2.5336	5.2996	-839760.69		
9	0.1123	0.0154	0.7062	2.4900	-2.4831	5.2759	-835407.29		
10	0.1129	0.0156	0.7020	2.4898	-2.5003	5.2811	-839745.29		
11	0.1132	0.0159	0.6742	2.4879	-2.4497	5.2669	-797981.33		
12	0.1119	0.0161	0.6706	2.5007	-2.4451	5.2571	-837291.42		
13	0.1110	0.0162	0.7114	2.4902	-2.4846	5.2703	-836692.33		
14	0.1108	0.0164	0.6956	2.5005	-2.4632	5.2532	-837806.63		
15	0.1115	0.0164	0.6959	2.4941	-2.4759	5.2653	-839684.07		
16	0.1102	0.0167	0.7174	2.4897	-2.4858	5.2666	-839740.91		
17	0.1098	0.0169	0.7146	2.4886	-2.4609	5.2562	-835852.76		
18	0.1097	0.0170	0.7074	2.4942	-2.4652	5.2548	-839148.15		
19	0.1100	0.0169	0.7038	2.4937	-2.4785	5.2627	-839318.45		
20	0.1097	0.0171	0.7038	2.4943	-2.4732	5.2592	-839736.74		

Surprisingly, the log-likelihood did not vary smoothly in *n* (Table 2). For example, we observed the overall best likelihood score for *n *= 11, while *n *= 10 had a comparatively poor likelihood score. We believe that the discontinuity in likelihood scores was caused by aliasing issues. A site’s RSA can be high or low relative to the range of RSA values within a bin. After a small change in the number of bins (for example from *n *= 10 to *n *= 11), some sites that previously had a relatively low RSA for their bin will now have a relatively high RSA or vice versa. If those sites are particularly variable or particularly conserved, the change in their location relative to the bin center can substantially affect the quality of the model fit. For this reason, we do not think that it is reasonable to select the number of bins based on the likelihood score of the model. Instead, we opted for using a relatively large bin number (*n *= 20), which more accurately approximates a smooth dependency of model parameters on RSA.

### GY94 model provides a better model-fit than MG94 model

The GY94 model describes evolutionary rates using the two parameters *t* and *ω*. An alternative model, the Muse–Gaut model (MG94 [25]), uses instead the parameters *α* and *β*. The parameter *α* in MG94 corresponds to *t* in GY94 and the parameter *β *in MG94 corresponds to *tω *in GY94. If we fit a model without site variability (all parameters are constant across sites), the MG94 model and the GY94 model are identical. However, when we allow for site variability, the two models become different. The GY94 model is usually set up with a constant *t* and a variable *ω*[26,27]. This set-up implicitly assumes that the synonymous rate is constant across sites whereas the nonsynonymous rate is variable. The MG94 model, on the other hand, has been used to explicitly model both nonsynonymous and synonymous site variability [28].

Here, we have allowed both *ω *and *t* to vary with RSA, so we have considered both nonsynonymous and synonymous rate variation. However, in using the GY94 model, we have assumed that the two quantities that vary linearly with RSA are the synonymous rate and the ratio of the nonsynonymous to synonymous rates. *A priori*, it is just as reasonable to assume that the synonymous rate *α* and the nonsynonymous rate *β* are linear functions of RSA. In this case,the ratio *ω *=* β*/*α *would of course not be linear in RSA.

**Figure 3:**
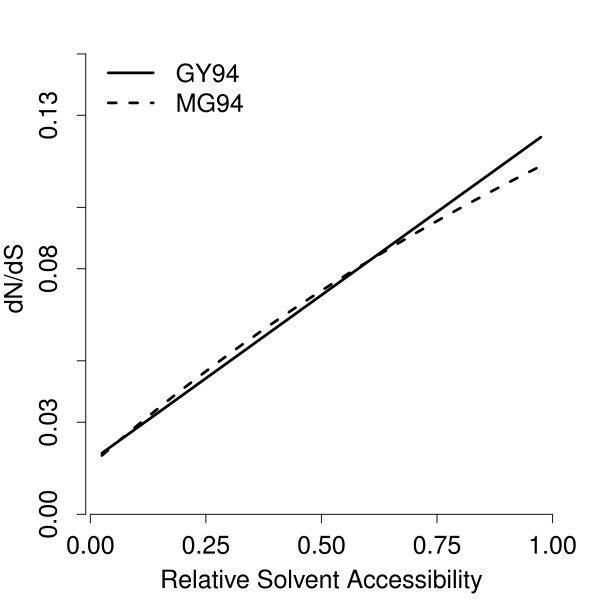
**Comparison of the GY94 and the MG94 models.** The solid line shows *ω *=* dN*/*dS *versus RSA, as estimated by the GY94 model. The dashed line shows the same for the MG94 model. Under the MG94 model, *ω *shows moderate curvature. The GY94 model provides a better fit to the data (ΔAIC = 14).

To assess whether the nonsynonymous rate *β *or the ratio *ω *=* β*/*α* is linear in RSA, we fitted a model in which *α*and *β* were linear functions of RSA. (*κ* was estimated per-bin). The resulting relationship of *ω *vs. RSA was similar but not identical to the one observed for linear *ω* (Figure 3). The log-likelihood score for this model fit was −839720.75, compared to a log-likelihood score of −839713.86 for the model with linear *ω*. The two models are not nested, so we cannot compare them using a likelihood ratio test. However, they are comparable via AIC, and the model with linear *ω *was clearly better (ΔAIC = 14).

### Effect of relative solvent accessibility on synonymous and nonsynonymous substitution rates

The previous subsections have shown that substitution rates at both synonymous and nonsynonymous sites are affected by RSA, and that the ratio *ω *=* dN*/*dS* changes linearly with RSA. If *ω *is linear in RSA and both *dN* and *dS* vary with RSA, then we expect *dN* and *dS* individually to not be linear in RSA.

The quantities *dN* and *dS* are not parameters that are estimated in the model fit. Instead, they are derived quantities that we can calculate once the model has been fit to the data. One complication in calculating *dN* and *dS* arises, however: There are multiple definitions of these parameters. For example, *dS* is defined as the number of synonymous differences divided by the number of synonymous sites in the sequence. We obtain the number of synonymous differences by summing over appropriate elements in the matrix *Q*[29]. The number of synonymous sites can be obtained in two different ways. First, we can simply count the number of sites atwhich a mutation would lead to a synonymous change, using fractional counts for sites at which mutations can cause either a synonymous or a nonsynonymous change. This method of counting gives us the physical-sites definition of *dS*[30]. Second, we can weigh each site with the probability that a synonymous mutation will occur at this site under the fitted model. This method of counting sites gives us the mutational-opportunity definition of *dS*[30]. The same two definitions exist for *dN*.

**Figure 4:**
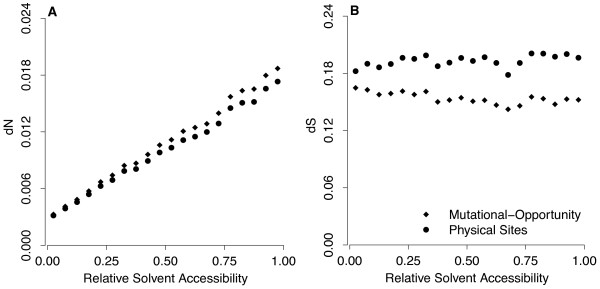
**Evolutionary rates *dN *and *dS*.** (**A**) The nonsynonymous rate, *dN*, correlates strongly with RSA under both the mutational-opportunity definition and the physical-sites definition. (**B**) The synonymous rate, *dS*, shows a moderate negative correlation with RSA under the mutational-opportunity definition and no slope under the physical-sites definition. The fitted model had linear *ω*, linear *t*, and per-bin *κ*.

The mutational-opportunity and the physical-sites definitions gave nearly identical results for *dN* (Figure 4A). In both cases, *dN* showed a strong increasing trend with RSA, with a slight deviation from linearity for higher RSA values. By contrast, the two definitions gave somewhat different results for *dS*. Under the mutational-opportunity definition, *dS* was decreasing with RSA, whereas under the physical-site definition it showed no obvious trend (Figure 4B).

### The effect of core size and expression level on evolutionary rate

In yeast, the primary determinant of evolutionary rate is gene expression level [31,32]. A second determinant is protein structure, measured either by contact density [7] or by core size [9]. Thus, we investigated how the slope and the intercept of the linear function *ω *=* ω*_0_ + *ω*_1_*r *changed with protein core size (measured by average RSA) and with gene expression level (measured by mRNA abundance).

**Figure 5:**
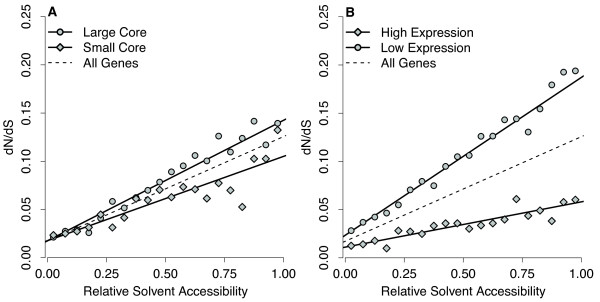
**Dependency of *ω *=* dN/dS *on protein core size and expression level.** (**A**) Core size affects evolutionary rate on the surface of the protein but not in the core. (**B**) Expression level affects evolutionary rate both on the surface and in the core. However, it has a bigger effect on the surface of the protein. In both figures, the solid lines were estimated jointly from the data using a linear dependency of *ω *on RSA. Points for individual bins are shown for illustration purposes only. They were estimated using a per-bin model for *ω*. The dashed black line represents the genome-wide trend, as shown in Figure 2, and is provided as a reference.

Franzosa and Xia showed that the slope of *ω *changed with core size while the intercept remained nearly unchanged. We repeated their analysis by identifying the proteins with the 33% largest and smallest cores and fitting a joint evolutionary model to these proteins. We fitted one line each for *κ* and *t* but fitted two separate lines for *ω*, one for the large-core proteins ( $\omega^{\text{lc}}=\omega_{0}^{\text{lc}}+\omega_{1}^{\text{lc}}r$) and one for the small-core proteins ( $\omega^{\text{sc}}=\omega_{0}^{\text{sc}}+\omega_{1}^{\text{sc}}r$), as shown in Figure 5. We found that small-core proteins displayed a smaller slope than large-core proteins ( $\omega_{1}^{\text{sc}}=0.082$ vs. $\omega_{1}^{\text{lc}}=0.127$). This difference in slopes was significant (likelihood ratio test, *P *= 6.41×10^−9^). By contrast, the intercepts were not significantly different (likelihood ratio test, *P *= 0.136), and we found ( $\omega_{0}^{\text{sc}}=\omega_{0}^{\text{lc}}=0.018$).

The two slopes we found were more similar to each other than the ones found by Franzosa and Xia [9]. The main difference between our data set and theirs was that we used more stringent criteria to match sequences to structures. To verify that we could reproduce the results of Ref. [9], we relaxed our criteria for alignment length to 70%, thereby increasing our dataset to 870 sequence-structure pairs. For this larger data set, we found a similar slope for large-core proteins as found before ( $\omega_{1}^{\text{lc}}=0.124$), but the slope for small-core proteins was reduced ( $\omega_{1}^{\text{sc}}=0.058$). These slopes were consistent with the findings of Ref. [9].

We carried out a similar analysis on high-expression and low-expression genes, fitting a separate line to each group of proteins ( $\omega_{1}^{\text{he}}=\omega_{0}^{\text{he}}+\omega_{1}^{\text{he}}r$ for high-expression genes, $\omega_{1}^{\mathrm{le}}=\omega_{0}^{\mathrm{le}}+\omega_{1}^{\mathrm{le}}r$ for low-expression genes). We found a substantial difference in slope between these two groups of genes ( $\omega_{1}^{\text{he}}=0.047$ vs. $\omega_{1}^{\mathrm{le}}=0.164$). The difference was significant (likelihood ratio test, *P *= 1.75×10^−62^). We also found a difference in intercept ( $\omega_{0}^{\text{he}}=0.011$ vs. $\omega_{0}^{\mathrm{le}}=0.023$) and this difference was significant as well (likelihood ratio test, *P *= 6.05×10^−12^). Similar results were found when we used codon adaptation index as a proxy for gene expression level (data not shown).

**Figure 6:**
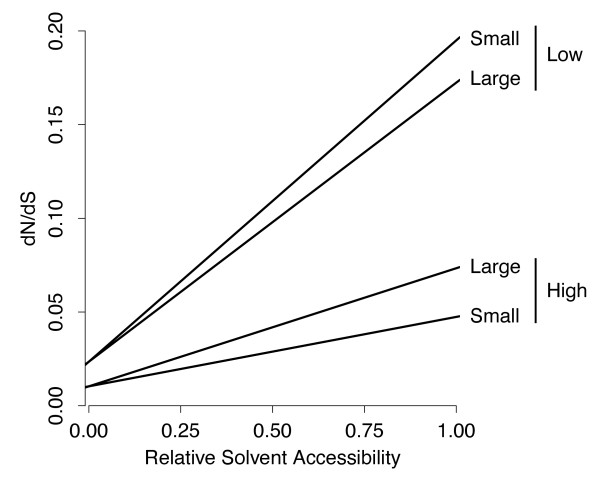
**Joint analysis of the effects of both core size (small or large) and expression level (low or high) on the relationship between *ω *=* dN/dS *and RSA.** Only the fitted lines are shown. Surprisingly, for low-expression genes, small-core proteins evolve faster than larger-core proteins. This relationship is reversed in a larger dataset obtained with less-stringent criteria (see text).

Finally, we carried out a joint analysis of core size and expression level by extracting four groups of proteins from our data set: proteins with (1) high expression level and large core, (2) high expression level and small core, (3) low expression level and large core, and (4) low expression level and small core. Figure 6 shows the resulting model fit. Clearly, expression level plays a larger role in determining evolutionary rate than core size. However, the model with core-size-dependent slope showed a better fit than a model in which the slope depended only on expression level (likelihood ratio test, *P *= 5.33×10^−4^). Surprisingly, the effect of core size on slope was reversed for high- and low-expression genes. For high-expression genes, proteins with larger core size showed a larger slope in *ω* than did proteins with smaller core size, consistent with prior results. By contrast, low-expression proteins with larger core size showed a smaller slope than did proteins with smaller core size. However, this unexpected pattern disappeared when we repeated the above analysis on our expanded data set with 870 sequence-structure pairs. There, the large-core-size proteins had the larger slope in all cases, consistent with prior results (data not shown).

## Discussion

We have developed a method that models the evolutionary rate of a coding sequence within the context of the protein’s 3-dimensional structure. Our method is a simple extension of the standard GY94 model, modified such that all parameters are functions of relative solvent accessibility (RSA). We have found that the evolutionary-rate ratio *ω *=* dN*/*dS*, the branch length *t*, and the transition–transversion bias *κ *all depend on RSA. The overall best fitting model had a linear relationship of *ω* and *t* with RSA, while *κ *showed small deviations from strict linearity. In the second-best model, all parameters had a linear relationship with RSA.

Our method presents a unified statistical framework for comparing RSA-dependent model parameters among different groups of proteins. Using this framework, we have shown that protein core size affects only the slope of *ω* as a function of RSA, but not the intercept. The most buried residues have—on average—the same *ω *value regardless of protein core size. By contrast, expression level affects *ω* even for the most buried residues.

We have found that the variation in *ω* with RSA is substantial; for the most exposed residues, *ω *was on average 5-10 times larger than it was for the most buried residues. This observation highlights the importance of incorporating protein structure into models of coding-sequence evolution. Traditional models of rate variation [27,29,33] cannot distinguish between rate variation caused by protein structure and rate variation caused by other factors (e.g., positive or negative selection on sites of functional importance). As an obvious extension to the work presented here, we can combine the present model with more traditional models of rate variation by allowing for additional rate variation among sites with similar RSA. This work will be presented elsewhere [34].

Our findings here are broadly consistent with the findings of Franzosa and Xia [9]. We have confirmed the linear relationship between *dN*/*dS *and RSA in an independently derived data set; we have also confirmed that proteins with larger core size show a faster increase of *dN*/*dS *with increasing RSA than proteins with smaller core size. Our work goes beyond Franzosa and Xia’s findings by demonstrating that the evolutionary rate of fully buried residues is independent of protein core size, that expression level affects evolutionary rate at all RSA values, and that the GY94 model provides a better model fit than the MG94 model when RSA-dependent evolutionary rates are considered. Our work also suggests that nucleotide-level processes vary systematically with protein structure.

In our joint analysis of core size and expression level, we made the unexpected observation that the effect of core size on the slope of *ω *is reversed for genes with low expression level. However, this observation disappeared in a larger data set obtained under slightly less stringent criteria for matching sequences to PDB structures. We can offer no good explanation for this observation. It could be a statistical fluke. The number of genes in each of the four groups (low expression and small core size, low expression and large core size, high expression and small core size, high expression and large core size) is relatively small in this analysis, so a few unusual proteins could skew the analysis. What exactly is the cause of this unexpected observation may have to be clarified in future analyses, either using expanded data sets—as more structures become available—or using data from different organisms.

Our approach is conceptually related to other recent works attempting to combine protein structure with sequence evolution [10-13]. These works imposed structural constraints on sequence evolution via sophisticated energy functions describing how protein fold stability changes as amino acids are replaced. In comparison, our approach is much more simplistic. However, we believe that this simplicity has substantial benefits. First, our approach is simple and fast. All the models we have used here can be fit within 10–15 minutes on an off-the-shelf laptop. Second, our approach yields results that can be interpreted easily. Instead of a single *ω* value per gene, we obtain two values, an intercept and a slope. The intercept tells us to what extent selection constrains the most buried residues; the slope tells us by how much selection relaxes as we move towards more exposed residues. Third, our approach can be implemented with relative ease in existing modeling frameworks such as HyPhy [22].

Following Franzosa and Xia [9], we used a model that fit a single rate ratio *ω*, regardless of which amino acids were substituted into which other ones. A recent study has shown that such models can always be improved upon with amino-acid dependent transition rates, even if amino acids are grouped into exchangeability categories at random [35]. This finding is not entirely surprising, considering that amino-acid substitution matrices have consistently been found to depend substantially on the amino-acid identity (e.g. Refs. [36-38]). Therefore, it would be desirable to develop codon-level substitution models that accurately capture this rate variation, without adding too many additional parameters. Approaches that have been suggested include automatically grouping amino acids into exchangeability categories [39,40] and decomposing amino-acid substitution rates into components corresponding to biophysical properties of amino acids (LCAP model, Ref. [41]). Yet substitution rates also depend on protein structure [1,2,6,42], and thus one would want to incorporate structure into these models as well. One study developed a variant of the LCAP model where parameters were fit separately to buried and exposed sites and found to be significantly different [17]. Since we have seen here that substitution rates seem to depend continuously (and linearly) on RSA, it might be worth it to investigate a variant of the LCAP model in which rate parameters are linear functions of RSA. Such a model would have the same number of parameters as the model in Ref. [17] but would quite possibly provide a better fit to the data. Alternatively, one could attempt to incorporate an RSA-dependence into models that automatically group amino acids [39,40].

We found that in our model, both *t* and *κ *varied with RSA. We believe that this finding reflects the effect of selection on nucleotide-level processes. First, equilibrium amino-acid frequencies vary with RSA [20,43], and this variation will have some effect on equilibrium codon frequencies. Second, protein structure also seems to exert a direct selection pressure on synonymous codon choice [44-50], most likely through an interaction between the translation process and protein folding. A more realistic model could represent this relationship between protein structure and the nucleotide-level substitution process more accurately, for example via a structure-dependent variant of the FMutSel model [51] or by extending models such as the LCAP model [17,41] to contain structure-dependent terms for nucleotide-level processes.

The challenge in developing any such models will be to make them realistic yet sufficiently simple so they can be fit to moderately sized data sets. An alternative, simpler strategy could be to calculate equilibrium codon frequencies in an RSA-dependent manner. We considered calculating codon frequencies per bin and found that doing so generally improved AIC scores but did not eliminate the need for RSA-dependent *t* or *κ*, nor did it alter any of our other results in a substantive way (not shown).

Our method requires a solved crystal structure to calculate RSA values. Although the Protein Data Bank (PDB) has been growing rapidly over the past decade, the number of available structures is still small compared to the number of available sequences. For example, many of the yeast sequences we used in our analysis did not have a corresponding structure. For those sequences, we relied on homologous protein structures solved in related organism. Homology mapping performs relatively well in predicting relative solvent accessibility [49] but clearly it is not perfect. Further, certain proteins or regions of proteins, such as membrane proteins or intrinsically disordered regions, can usually not be crystalized. Thus, our method cannot be applied to such proteins or regions of proteins.

Our method assumes that RSA remains constant throughout evolution. Yet every amino-acid replacement will cause some distortion in the protein structure [52], and RSA values at homologous sites will slowly diverge with increasing sequence divergence [49]. In the future, if either the number of available PDB structures increases drastically or if atom-level computational modeling of protein structures becomes sufficiently reliable, we will able to study how changes in structure correlate with evolutionary rate.

## Conclusions

Our work has shown that the evolutionary rate ratio *ω*, the branch length *t*, and the transition–transversion bias *κ *all vary significantly with relative solvent accessibility (RSA). All three parameters show an approximately linear RSA dependency. In general, both the slope and intercept of the *ω*–RSA dependency differ according to the specifics of individual genes, such as protein structure and gene expression level. Our work demonstrates that protein structure can be an important ingredient in comparative sequence analysis. Our work further suggests that a tighter integration of structural and sequence data will improve the performance of comparative analysis methods.

## Methods

### Homology mapping and categorization of genes

In order to construct a large data set of sequences with corresponding structures, we obtained open reading frames (ORFs) of the yeast *Saccharomyces cerevisiae* from the Saccharomyces Genome Database [53] and aligned them with orthologous *Saccharomyces paradoxus* sequences using MUSCLE [54]. Each ORF was translated and searched against the Protein Data Bank (PDB) [55] using the PSI-BLAST algorithm [56] and then paired with the structural chain with the lowest alignment E-value. To ensure that enough of the yeast protein was represented in the chain and that the PDB structure was a reasonable homology model, we only considered pairs with > 80*%* alignment length and > 40*%*sequence identity for analysis. Our final data set had 587 sequence–structure pairs. A data set with relaxed criteria used > 70*%* alignment length and > 40*%*sequence identity. This data set had 870 sequence–structure pairs.

The percent solvent-accessible surface area (ASA) for each aligned residue was calculated using DSSP [57]. We obtained relative solvent accessibility (RSA) by normalizing ASA values with the surface areas of an extended Gly-X-Gly peptide [58].

### Calculation of evolutionary rates

The codons from the yeast alignments were binned by the RSA value of their respective residues, as described [9]. Protein core size was estimated by the average RSA value over all residues in a protein. We considered a structure to have a large core if its average RSA ranked within the bottom third of all average RSA values and to have a small core if ranked within the top third of all average RSA values [9]. Yeast expression data measured in mRNA abundance per cell was obtained from [59]. Codon adaptation index (CAI), a measure of the strength of codon usage bias, was used as an alternative for expression level, since the latter may be biased by laboratory growth conditions of the yeast cells [60]. Both expression level and CAI were ranked and divided into thirds with the top third representing high-expression genes and the bottom third low-expression genes.

We implemented the model described by Eq. (1) in the HyPhy batch language [22]. We estimated codon frequencies (*Π*_*j*_) using F3×4 model.

We calculated synonymous (*dS*) and nonsynonymous (*dN*) substitution rates according to the mutational-opportunity and the physical-sites definitions, as described [29,30].

### Statistical analysis

We used the Akaike information criterion (AIC) [23,24] to rank models by their quality of fit. For pairwise comparison of nested models, we also carried out likelihood-ratio tests. All statistical analyses were performed using the statistics software R [61].

## Abbreviations

AIC: Akaike information criterion; CAI: Codon adaptation index; GY94: Goldman-Yang 1994; HyPhy: Hypothesis testing using Phylogenies (software); MG94: Muse-Gaut 1994; ORF: Open reading frame; PDB: Protein data bank; RSA: Relative solvent accessibility.

## Authors’ contributions

MPS collected data, developed HyPhy batch files, ran analyses, prepared figures, and wrote the manuscript. AGM developed HyPhy batch files. COW conceived of the study, participated in its design and coordination, and wrote the manuscript. All authors read and approved the final manuscript.
